# Proportions of four distinct classes of sensory neurons are retained even when axon regeneration is enhanced following peripheral nerve injury

**DOI:** 10.3389/fnana.2023.1303888

**Published:** 2023-11-06

**Authors:** Samia Khan, Dario I. Carrasco, Robin Isaacson, Arthur W. English

**Affiliations:** Department of Cell Biology, Emory University School of Medicine, Atlanta, GA, United States

**Keywords:** enhancing axon regeneration, peripheral nerve injury, dorsal root ganglion cell classes, compound 11, VGLUT1, TRPV1, IB4, tyrosine hydroxylase

## Abstract

**Introduction:**

Recovery from peripheral nerve injuries is poor because axon regeneration is slow and inefficient. Experimental therapies that increase signaling of neuronal brain-derived neurotrophic factor (BDNF) through its TrkB receptor or through its downstream effectors enhance axon regeneration, increasing the number of motor and sensory neurons whose axons successfully regenerate and reinnervate muscle targets. The goal of this study was to compare the proportions of four different classes of sensory (dorsal root ganglion, DRG) neurons that successfully reinnervate two different muscle targets in control mice and mice treated pharmacologically to enhance axon regeneration.

**Methods:**

Following sciatic nerve transection and repair, C57BL/6 J mice were treated for 2 weeks, either with R13, a prodrug that releases the small molecule TrkB ligand, 7,8-dihydroxyflavone, with compound 11 (CP11), an inhibitor of asparaginyl endopeptidase (δ-secretase), or with a control vehicle. Four weeks after injury, different fluorescent retrograde tracers were injected into the gastrocnemius and tibialis anterior muscles to mark DRG neurons that had successfully reinnervated these muscles. Using immunofluorescence, retrogradely labeled DRG neurons also expressing markers of four different sensory neuronal classes were counted.

**Results and discussion:**

Treatments with R13 or CP11 resulted in muscle reinnervation by many more DRG neurons than vehicletreated controls, but neurons expressing proteins associated with the different classes of DRG neurons studied were largely in the same proportions found in intact mice.

## 1. Introduction

The reason most often ascribed to poor recovery from peripheral nerve injuries (PNIs) is the slow and ineffective process of axon regeneration. By enhancing this process, the effectiveness of axon regeneration could be improved and heighten functional recovery. Activity-dependent experimental treatments for PNIs, such as brief low frequency (20 Hz) electrical stimulation (Al-Majed et al., 2000) or exercise (Sabatier et al., 2008; Udina et al., 2011), have been developed, but they are not viable for a significant number of individuals because of the nature of their nerve injuries (Park et al., 2019; Maugeri et al., 2021). The effectiveness of these therapies relies on the promotion of increased signaling by brain-derived neurotrophic factor (BDNF) via its tropomyosin receptor kinase B (TrkB) receptor (Gordon and English, 2016). A known small-molecule BDNF mimetic, 7,8-dihydroxyflavone (7,8-DHF), signals effectively through the TrkB receptor. Systemic injections of 7,8-DHF following PNI promoted motor axon regeneration in mice (English et al., 2013). Oral administration of R13, a prodrug that is metabolized in the liver and gradually converts to 7,8-DHF (Chen et al., 2018), enhanced both motor and sensory axon regeneration following PNI (English et al., 2022). In R13-treated mice, axons of many more sensory neurons had regenerated and successfully reinnervated muscle targets than the vehicle-treated controls after sciatic nerve transection and repair.

We showed recently that asparaginyl endopeptidase (AEP), a lysosomal cysteine protease implicated in the pathology of Alzheimer’s disease (Zhang et al., 2014), inhibited axon regeneration in injured nerves (English et al., 2021). One of the known substrates of AEP is the axonal microtubule-associated protein, Tau. Tau is cleaved by AEP on the carboxyl sides of asparagine residues N255 and N368, thereby removing its microtubule-binding domain completely (English et al., 2021). During axon regeneration and elongation, an axonal cytoskeleton created from microtubules helps stabilize the nascent regenerating axons (Drubin and Kirschner, 1986). These microtubules are, in turn, stabilized by Tau (Black et al., 1996). Axon regeneration is enhanced if AEP is knocked out in the regenerating axons (English et al., 2021). Inhibition of AEP is a downstream target of TrkB signaling and treatments that increase TrkB signaling result in a decrease in active AEP at the site of nerve injury (English et al., 2021; Isaacson et al., 2023). Treatments with the specific AEP inhibitor, Compound 11 (CP11), resulted in an enhancement of motor and sensory axon regeneration similar to that observed after R13 treatments (Isaacson et al., 2023).

Several recent studies have demonstrated extensive cellular heterogeneity among dorsal root ganglion (DRG) sensory neurons. For example, using single nucleus RNA sequencing, nine different DRG neuron classes were defined (Renthal et al., 2020) (Table 1), each associated with the expression of a series of cell type-specific (CTS) genes. Following spinal nerve crush, transcription of CTS genes was decreased, and all injured neurons began expressing mRNAs for a common suite of genes related to axon regeneration, such as *Atf3* and *Sprr1a*. Following successful axon regeneration after nerve crush, transcription of the CTS genes was restored. Markers for these DRG classes thus form a template for analysis of whether experimental treatments for PNI, such as R13 or CP11, will promote the regeneration of different classes of sensory axons equally. This question might be especially appropriate in the case of peripheral nerve transection injuries, where misdirection of regenerating axons is well known and reinnervation of targets, such as individual muscles, might be via very different sets of neurons than their original innervation (Brushart, 1993, 2012; English, 2005; Gordon and English, 2016). The goal of this project was to investigate the effectiveness of these treatments on axon regeneration of selected classes of muscle sensory neurons after peripheral nerve transection and surgical repair.

**Table 1:** Dorsal root ganglion cell classes.

Class	Name	Gene identifier*	Protein marker used
PEP	Peptidergic nociceptors	*Tac1*	TrpV1
PEP1		*Gpx3*	
PEP2		*Trpm8*	
NP	Non-peptidergic nociceptors	*Mrgprd*	Isolectin B4
NF	Large neurofilament	*Nefh*	
NF1		*Slc17a7*	VGLUT1
NF2		*Pvalb*	
NF3		*Ntrk2*	
TH	Tyrosine hydroxylase	*Th*	TH
SST	Somatostatin	*Nppb*	

^*^After Renthal et al. (2020).

## 2. Methods

### 2.1. Animal surgeries

All experimental methods used were approved by the Institutional Animal Care and Use Committee of Emory University (PROTO201800101). In all experiments, C57BL/6 J wild-type mice were used. Dorsal root ganglia from four mice (all 6–12 weeks old, two male and two female) in each of four groups of mice were studied: intact, R13-treated, CP11-treated, and vehicle-treated. Intact mice did not undergo any surgery, but in all other groups, mice were isoflurane-anesthetized and the right sciatic nerve was exposed in the mid-thigh, cut with sharp scissors, and immediately repaired by simple end-to-end anastomosis. Repaired nerve segments were secured in place using 6 μL of fibrin glue (Akhter et al., 2019). On the third day following surgery, mice began oral treatments with either R13 (21.8 mg/kg) or the vehicle in which it was prepared (5% DMSO/0.5% methylcellulose). CP11-treated mice received intraperitoneal (i.p.) injections (10 mg/kg). All treatments were repeated daily, 5 days per week, for 2 weeks. R13 was obtained from Sundia MediTech, Shanghai, China (Lot No: A0257-10014-16). CP11 was purchased from Santa Cruz Biotechnology (catalog # sc-319780).

**Figure 1:**
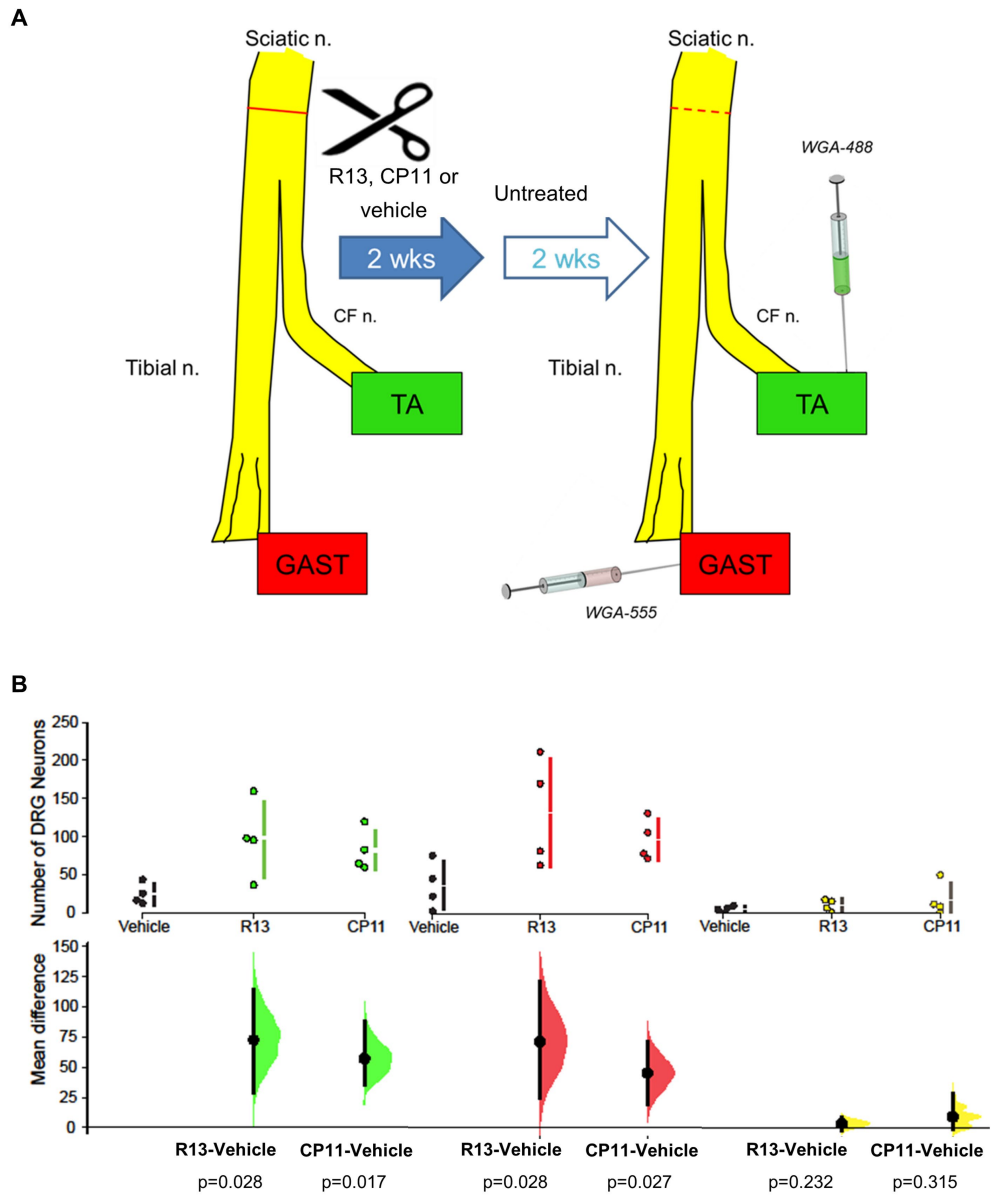
**(A)** Diagram of experimental protocol. Adult mouse sciatic nerves were cut and surgically repaired. Beginning 3 days later, mice were treated with R13, CP11, or the vehicle that they were dissolved in, 5 days per week for 2 weeks. Two weeks after the cessation of these treatments, different retrograde fluorescent tracers were injected into the tibialis anterior (TA) and gastrocnemius (GAST) muscles to mark the sensory (DRG) neurons that had successfully regenerated and reinnervated those muscles. For comparison, tracers were injected into the same muscles of a group of intact mice. **(B)** Mean (± SD) numbers of L4 DRG neurons containing the tracer injected into TA, GAST, or neurons containing both tracers in the different treatment groups in the present study are shown in the top row of graphs. In the bottom row, the results of 5,000 bootstrap samples of the original data are presented. Mean differences in the numbers of labeled neurons for the R13 and CP11 treatment groups from those in the vehicle-treated group are shown, as estimation frequency distributions. Mean differences are represented by dots, 95% confidence intervals by the ends of the vertical error bars. The *p* values resulting from two-sided permutation t-tests are shown below each comparison.

For all mice, 4 weeks after the nerve repair surgery, and 2 weeks after the end of treatments, wheat germ agglutinin (WGA), conjugated to different fluorophores, was injected into the lateral and medial heads of the gastrocnemius (GAST) muscle (WGA-555), and the tibialis anterior (TA) muscle (WGA-488). The TA is a flexor muscle innervated by axons coursing in the common fibular branch of the sciatic nerve. In contrast, GAST is an extensor and innervated by axons coursing in the tibial branch. Both muscles have a similar fiber type composition. During recovery from sciatic nerve transection and repair, significant misdirection of regenerating axons innervating these muscle targets was shown, resulting in their functionally inappropriate reinnervation (English, 2005). This misdirection was exacerbated if axon regeneration was stimulated using low frequency electrical stimulation (English, 2005). Injections were made using a Hamilton syringe with a 28G needle, with 2 μL of tracer per muscle (Figure 1). These tracers were taken up and transported retrogradely and marked the somata of sensory DRG neurons that innervated or had successfully reinnervated these muscles. Three days later, the mice were euthanized by intraperitoneal injection of Euthasol (pentobarbital sodium and phenytoin sodium, 150 mg/kg) and perfused transcardially with saline followed by 4% paraformaldehyde, pH 6.9. The L4 DRGs were harvested and cryoprotected in a 20% sucrose solution at least overnight before sectioning.

### 2.2. Identification of markers of neuronal classes in regenerated sensory neurons

Cryostat sections of the harvested L4 dorsal root ganglia, cut at 40 μm thickness, were reacted with different reagents to evaluate the expression of proteins associated with four different classes of DRG neurons reinnervating the GAST and TA muscles. Binding of isolectin B4 (IB4) or reaction to a set of primary and secondary antibodies was used to mark the different proteins studied (Table 2). Immunoreactivity to the vesicular glutamate transporter 1 (VGLUT1) marked one subclass (NF) of neurons characterized by the expression of the high molecular weight neurofilament protein. Binding of an antibody to the transient receptor potential cation channel subfamily V member 1 (TrpV1) was used to identify peptidergic nociceptors (PEP). The binding of IB4 was used to identify non-peptidergic (NP) unmyelinated primary afferent neurons. Immunoreactivity to tyrosine hydroxylase (TH) marked C-low threshold mechanoreceptor afferents. Antibodies to VGLUT1 and TH were combined on one set of sections and anti-TrpV1 and IB4 were used in combination on an alternate set of sections. Serial DRG sections were placed alternately onto charged glass microscope slides and incubated with a 1X phosphate-buffered saline (PBS) solution containing 10% bovine serum albumin (BSA) and 0.5% Triton on the slides for 1 hour. The slides were then incubated at room temperature with primary antibodies overnight and washed with 1X PBS three times before application of secondary antibodies or IB4 for 2 hours and then coverslipped using Vectashield®. All sections made through the ganglia were used for study.

**Table 2:** Antibodies used, with dilutions.

Protein	Primary antibody	Secondary antibody/lectin
VGLUT1	Synaptic systems AB 135304 (1:1000)	Alexa Fluor 647- Goat anti-guinea pig (1:500)
TH	Millipore AB152 (1:200)	Alexa Fluor 405- Goat anti-rabbit (1:200)
TrpV1	Invitrogen PA1-748 (1:100)	DyLight™405 Donkey anti-guinea pig (1:200)
Isolectin B4	Griffonia simplicifolia Isolectin B4, DyLight™ 649 (1:200)	

### 2.3. Imaging and image analysis

Images of sections were captured at 10x magnification using a Leica DM6000 upright fluorescence microscope and Hamamatsu low-light camera, using HCImage software. Sensory neurons that had regenerated successfully were identified both if the retrograde fluorescent label filled the soma and if a clear nuclear region devoid of the label was present. The neurons that reinnervated the GAST muscle were labeled with a red tracer (WGA-555), and the neurons that reinnervated the TA muscle were labeled with a green tracer (WGA-488). Some neurons were labeled with both tracers and appeared yellow. These cells were considered to have detected both tracers during reinnervation, as we have described previously (English, 2005; Isaacson et al., 2023), and analysis for these “Both” neurons was performed separately. Immunoreactivity to the proteins studied or IB4 lectin binding was captured using a third (UV, 350 nM) and a fourth (far red, 647 nM) filter set. For each of the four channels studied, the background fluorescence was subtracted and the mean gray value of five neurons subjectively identified as containing neither of the retrograde labels nor either of the markers of cell identity was measured using the FIJI software package. In subsequent analyses, the fluorescence intensity for each of the two protein markers of DRG classes was determined in each cell identified as a retrogradely labeled neuron. If this intensity was two standard deviations greater than the mean of the unmarked cells, the labeled neuron was scored as containing the marker protein. The proportion of all similarly retrogradely labeled neurons that were identified as expressing proteins associated with the different identified classes of DRG neurons studied was determined and compared between treatments. During all such observations, the persons doing the measurements were blinded to the treatment group under consideration.

### 2.4. Statistical analyses

The significance of differences in proportions of labeled DRG neurons assigned to different phenotypes was evaluated using estimation statistics to calculate effect sizes and their significance (Bernard, 2019; Ho et al., 2019). Proportions of DRG neurons of different phenotypes that were retrogradely labeled from GAST or TA, or contained both retrograde labels, were studied separately. In each treatment group in these three categories, 5,000 bootstrap samples were taken from our data samples, resulting in an effect size, which was reported as mean differences in each treatment group from the proportions found in intact mice, and bootstrap 95% confidence intervals. This tool also reported the results of two-sided permutation tests for each group. The *p* values included represent the likelihood of observing the calculated effect sizes if the null hypothesis of a zero difference from the proportion found in intact mice was true. A probability of less than 0.05 was considered a significant difference.

## 3. Results

### 3.1. R13 and CP11 administration enhance sensory axon regeneration

We counted the number of L4 DRG neurons that were retrogradely labeled from the TA and GAST muscles and the number of neurons containing both retrograde fluorescent tracers in mice treated with R13, CP11, or vehicle. These counts are summarized in Figure 1B. Consistent with results of our previous studies (English et al., 2022; Isaacson et al., 2023), treatments with either oral R13 or i.p. injected CP11 resulted in significantly increased numbers of L4 DRG neurons that had reinnervated the TA and GAST muscles, compared to vehicle-treated controls. The treatments did not produce a significant increase in the number of DRG neurons that contained both tracers.

### 3.2. Six different phenotypes of sensory DRG neurons were identified

**Figure 2:**
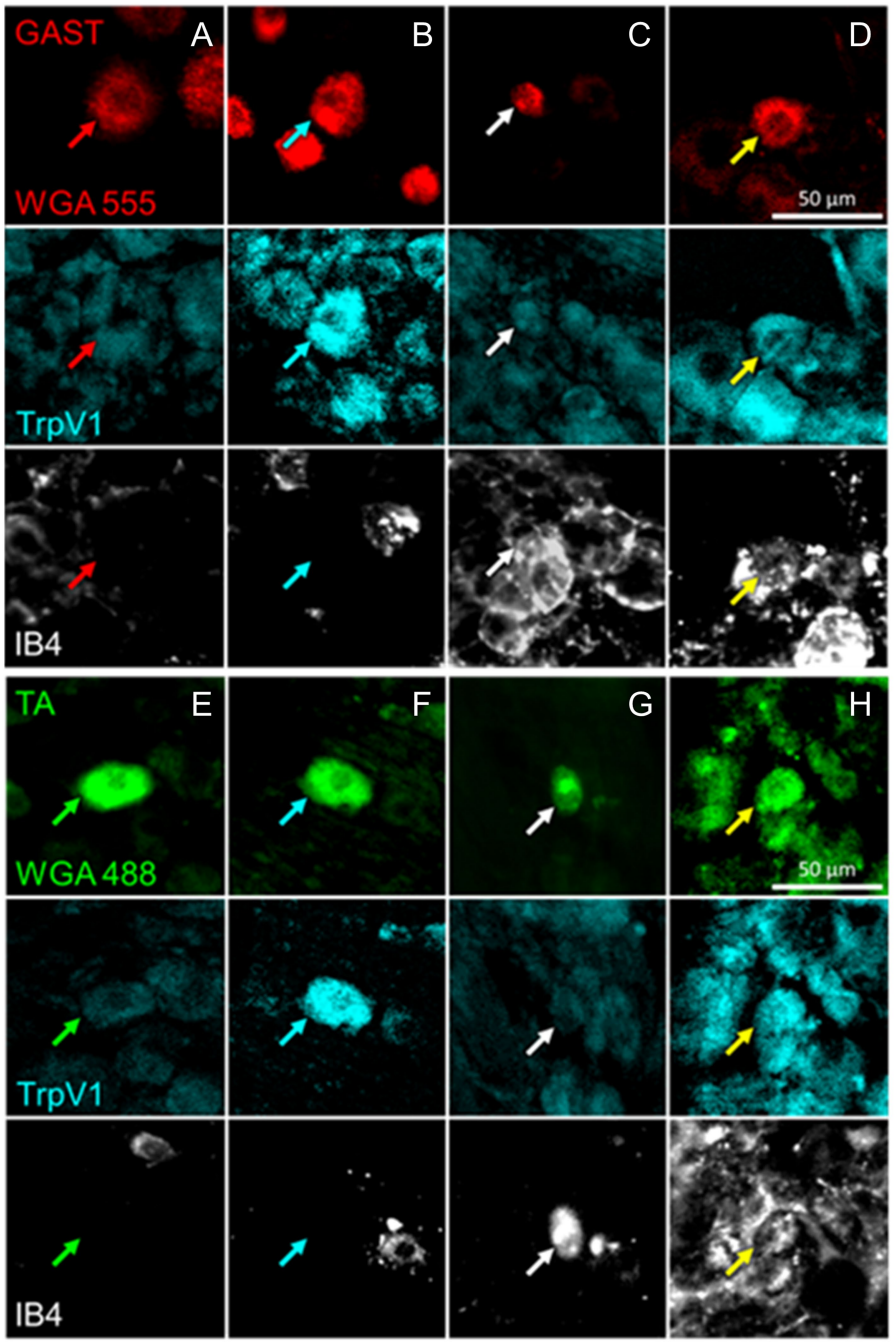
Examples of DRG neurons of different phenotypes, as defined by immunoreactivity (IR) to TrpV1, binding of isolectin B4 (IB4), and retrogradely labeled from the reinnervated gastrocnemius (GAST, columns **A-D**) or tibialis anterior (TA, columns **E-H**) muscles. In columns **A** and **E**, no IR to TrpV1 or IB4 binding is present in the cells marked by the red or green arrows. In Columns **B** and **F**, cells indicated by cyan arrows are IR for TrpV1 only. In columns **C** and **G**, white arrows point to cells binding IB4 only. In columns **D** and **H**, the cells identified by the yellow arrows are both IR to TrpV1 and bound IB4.

**Figure 3:**
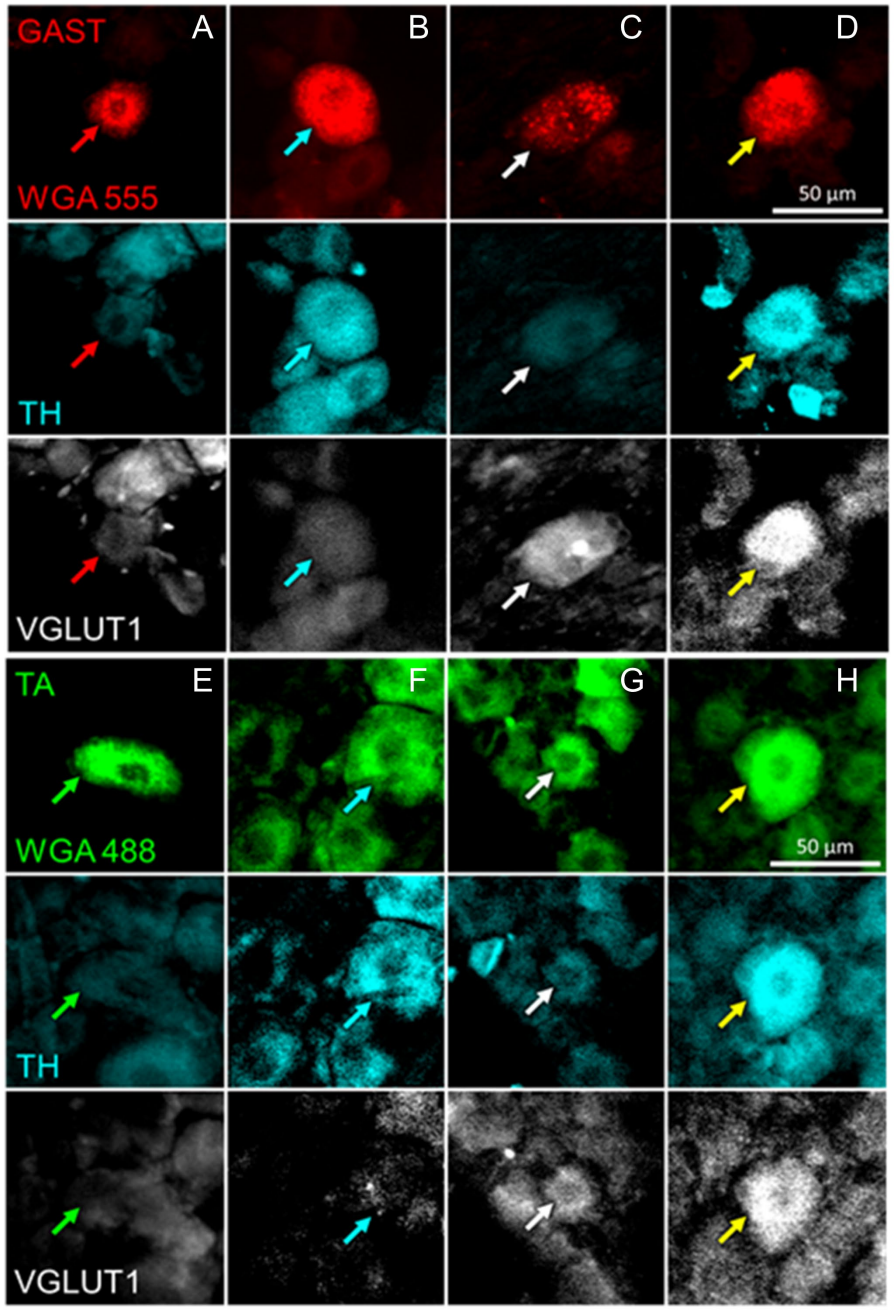
Examples of DRG neurons of different phenotypes, as defined by immunoreactivity (IR) to tyrosine hydroxylase (TH) and the vesicular glutamate transporter 1 (VGLUT1), and retrogradely labeled from the gastrocnemius (GAST, columns **A-D**) or tibialis anterior (TA, columns **E-H**) muscles. In columns **A** and **E**, no IR to either TH or VGLUT1 is present in the cells marked by the red or green arrows. In columns **B** and **F**, cells indicated by cyan arrows are IR for TH only. In columns **C** and **G**, white arrows point to cells IR to VGLUT1 only. In Columns **D** and **H** the cells identified by the yellow arrows are IR to both TH and VGLUT1.

Retrogradely labeled sensory neurons were analyzed to determine the proportions of cells expressing protein markers associated with four different classes of DRG neurons reinnervating the TA and GAST muscles. The combinations of antibodies raised in different species (Table 2) made possible the identification of neurons of six different phenotypes. Cells immunoreactive to VGLUT1 (NF1), TH (TH), and both VGLUT1 and TH were identified in one set of sections, and TrpV1 (PEP), binding IB4 (NP), or both were identified in the other set of histological sections. Examples of DRG neurons retrogradely labeled from GAST and TA that were scored as positive for TrpV1 immunoreactivity, IB4 lectin binding, or both are shown in Figure 2. Similar images of neurons immunoreactive to VGLUT1, TH, or both are shown in Figure 3.

Proportions of each of these six DRG neuron phenotypes, relative to the total number of retrogradely labeled DRG neurons encountered in the appropriate set of sections were determined. The significance of differences in proportions between treatment groups and the proportions found in intact mice was evaluated for each phenotype using bootstrap resampling of the differences in proportions between the three treatment groups (vehicle, R13, and CP11) and the intact control group. Mean differences and bootstrap 95% confidence intervals were determined. A treatment was judged to result in a significant change in proportions of the DRG neurons of the different phenotypes if results of a two-sided permutation test indicated a less than 5 % likelihood of observing the calculated effect sizes if the null hypothesis of a zero difference from the proportion found in intact mice was true.

#### 3.2.1. TrpV1+ neurons

**Figure 4:**
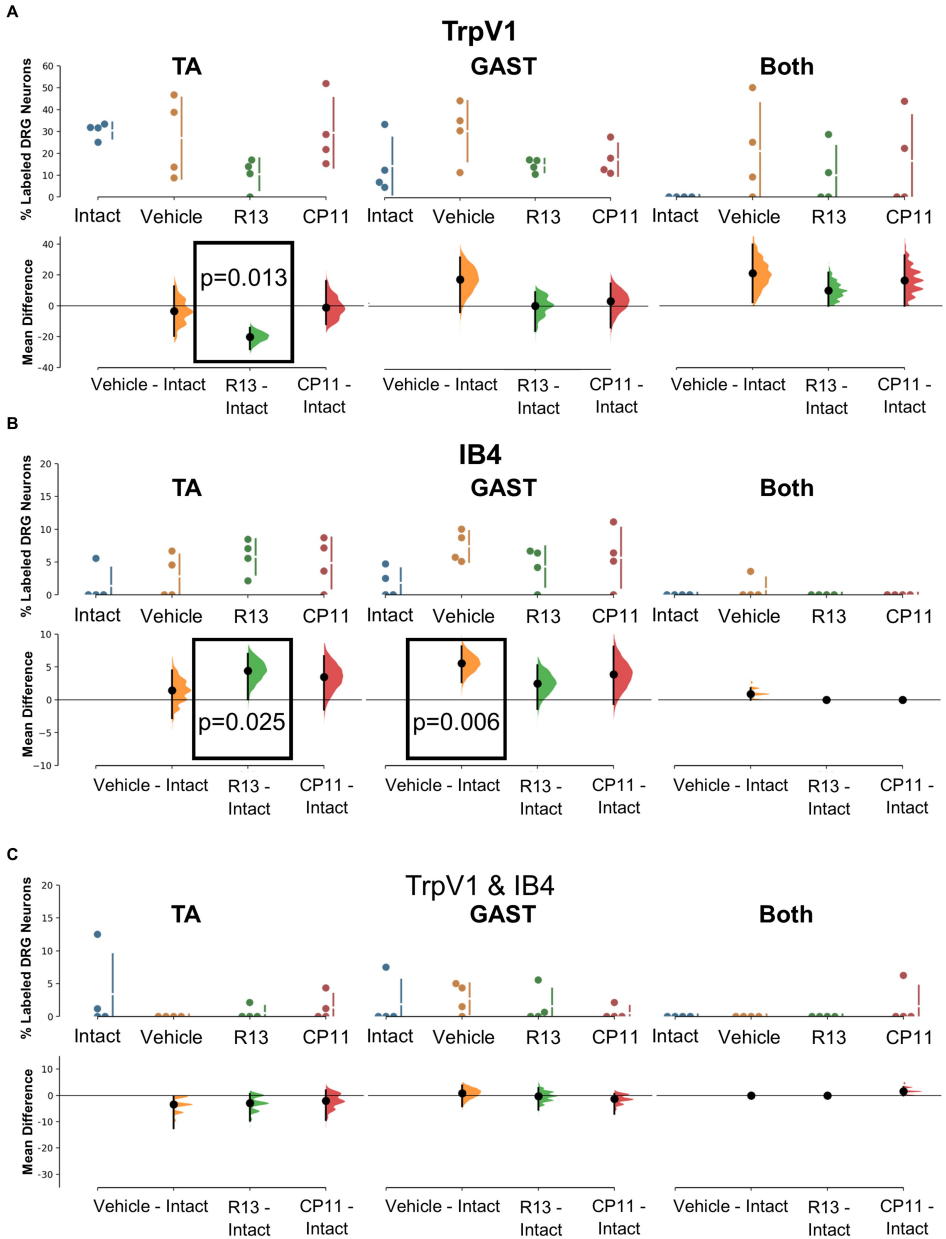
Results of analyses of proportions of L4 DRG neurons retrogradely labeled from TA, GAST, or containing Both retrograde tracers that were immunoreactive (IR) to TrpV1 **(A)**, positive for binding of isolectin B4 (IB4) **(B)**, or both TrpV1-IR and binding IB4 **(C)**. Each panel is a cumming estimation plot (Gardner and Altman, 1986). The top graph in each panel is a plot of the mean proportion (± SD) of neurons of the particular phenotype in intact mice and in mice 4 weeks after sciatic nerve transection and repair that had been treated for 2 weeks with vehicle, R13, or CP11. In the bottom graph in each panel, the results of 5,000 bootstrap samples of the original data are presented. Mean differences from intact for each of the treatment groups are shown, as estimation frequency distributions. Means are represented by dots and 95% confidence intervals by the ends of the vertical error bars. Boxes containing p values resulting from two-sided permutation *t*-tests are shown only when the likelihood that, based on the effect size observed, the null hypothesis of a zero difference from intact is true is less than 0.05.

Mean (±SD) proportions of labeled DRG neurons that were immunoreactive to TrpV1, a protein associated with peptidergic nociceptors (Cavanaugh et al., 2009), are shown in the top panels of Figure 4A for intact mice and mice in the different experimental treatment groups. Data were analyzed separately for neurons labeled from tracers injected into the TA and GAST muscles and for neurons containing both retrograde tracers (Both). Based on the results of bootstrap resampling, a significant decrease in the proportion of DRG neurons labeled from TA, but not GAST or Both, was determined for mice treated with R13 (Figure 4A: bottom panels). No other significant differences in proportions were found.

#### 3.2.2. IB4

The binding of fluorescent isolectin B4 was used to identify DRG neurons associated with non-peptidergic unmyelinated primary afferent neurons (Nagy and Hunt, 1982; Silverman and Kruger, 1990). Mean (±SD) proportions of labeled DRG neurons that bound IB4 are shown in the top panels of Figure 4B for intact mice and mice in the different experimental treatment groups. Significantly more DRG neurons that had reinnervated the TA muscle and bound IB4 were found in mice treated with R13 than in intact ganglia. Among DRG neurons reinnervating GAST, proportionally more bound IB4 in mice treated with vehicle than found in intact animals. All other comparisons were not statistically significant.

#### 3.2.3. TrpV1 and IB4 co-expression

A small subset of retrogradely labeled neurons was found to be both immunoreactive for TrpV1 and bound IB4, in both intact mice and in mice recovering from nerve injury. Mean (±SD) proportions of these TrpV1/IB4 DRG neurons are shown in the top panels of Figure 4C for intact mice and mice in the different experimental treatment groups. Using bootstrap resampling, no significant differences from the proportions found in intact mice were observed in any of the groups studied (Figure 4C: bottom panels). Treatments with R13 or CP11 did not result in a change in the proportion of these neurons.

#### 3.2.4. TH

**Figure 5:**
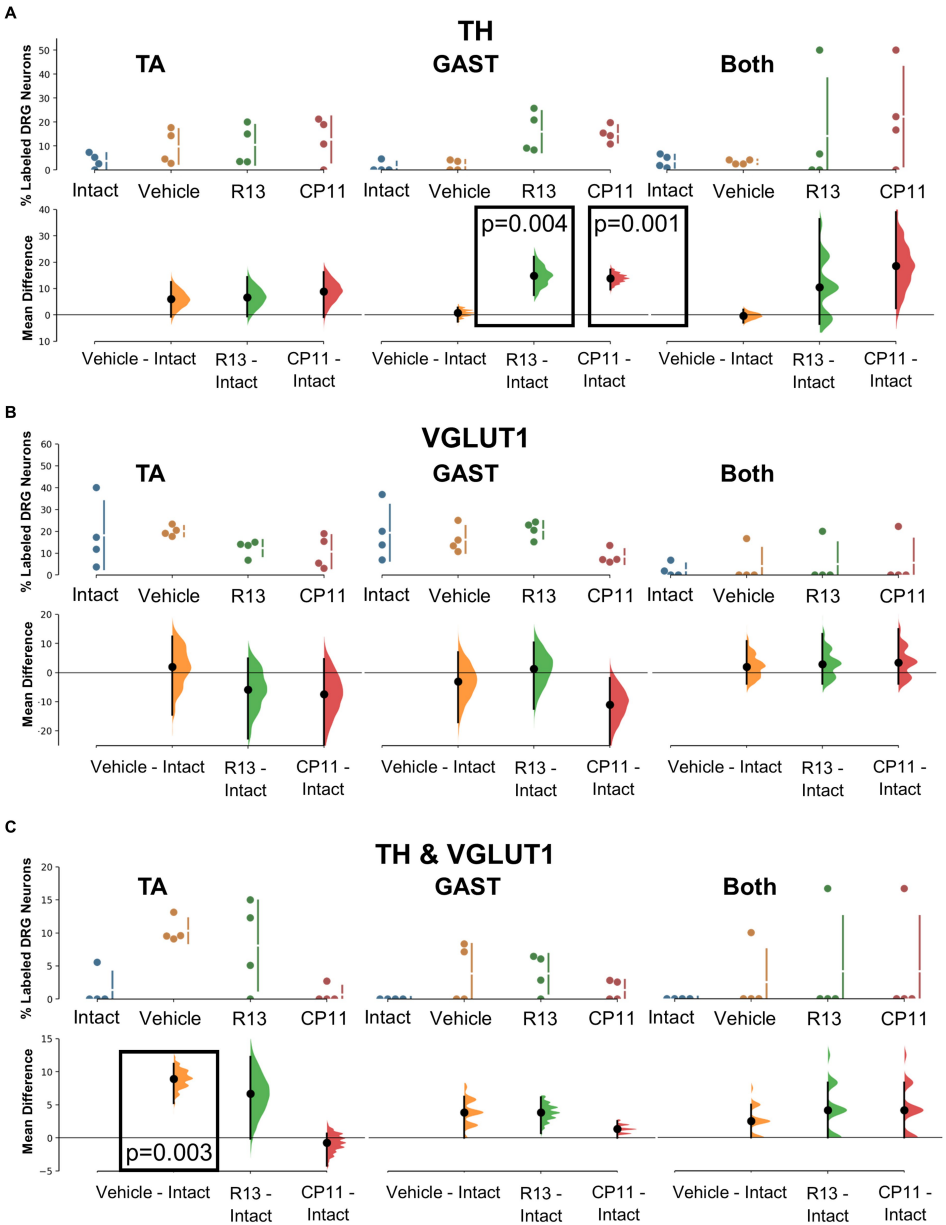
Results of analyses of proportions of L4 DRG neurons retrogradely labeled from TA, GAST, or containing Both retrograde tracers that were immunoreactive to TH **(A)**, VGLUT1 **(B)**, or both TH & VGLUT1 **(C)**. In each panel, the top graph is a plot of the mean proportion (± SD) of neurons of the particular phenotype in intact mice and in mice 4 weeks after sciatic nerve transection and repair that had been treated for 2 weeks with vehicle, R13, or CP11. In the bottom graph in each panel, the results of 5,000 bootstrap samples of the original data are presented. Mean differences from intact (± 95% confidence intervals) for each of the treatment groups are shown, as are estimation frequency distributions. Boxes containing *p* values resulting from two-sided permutation *t*-tests are shown only when the likelihood that, based on the effect size observed, the null hypothesis of zero difference is true is less than 0.05.

Tyrosine hydroxylase (TH) immunoreactivity in intact mice has been described in C-low threshold mechanoreceptors that innervate mainly skin (McGlone et al., 2014). We consistently found a small proportion (<10%) of DRG neurons retrogradely labeled from intramuscular injections of tracers that were also immunoreactive only for TH (Figure 3). Mean (±SD) proportions of these TH+ DRG neurons are shown in the top panels of Figure 5A for intact mice and mice in the different experimental treatment groups. Using bootstrap resampling, significant increases in the proportions of labeled TH-IR cells were found in both the R13- and CP11- treatment groups in neurons retrogradely labeled from GAST (Figure 5A: bottom panels). Treatments with either R13 or CP11 increased the proportion of successfully regenerating TH-IR muscle afferent neurons reinnervating GAST, but no significant effects of these treatments were found in neurons labeled from TA or those containing both retrograde tracers.

#### 3.2.5. VGLUT1

Primary afferent neurons expressing VGLUT1 are found in a subset of cells also expressing the high molecular weight neurofilament protein (Brumovsky et al., 2007). These neurons are associated with proprioceptive functions (Alvarez et al., 2004). Mean (±SD) proportions of retrogradely labeled L4 DRG neurons immunoreactive only to VGLUT1 are shown in the upper panels of Figure 5B for intact mice and mice in the different experimental treatment groups. No significant difference was found among the proportions of retrogradely labeled neurons expressing VGLUT1 reinnervating TA, GAST, or neurons containing both tracers (Figure 5B: bottom panels). Treatments with R13 or CP11 resulted in many more successfully regenerating DRG neurons than vehicle-treated controls but similar proportions of VGLUT1-IR neurons to those found in these control mice or intact mice.

#### 3.2.6. TH and VGLUT1 co-expression

A subset of L4 DRG neurons was found to be immunoreactive to both TH and VGLUT1. Mean (±SD) proportions of these TH/VGLUT1 neurons are shown in the upper panels of Figure 5C for intact mice and mice in the different experimental treatment groups. Using bootstrap resampling, we found that significantly more L4 DRG neurons that had reinnervated TA expressed these two proteins after vehicle treatment than intact mice, but not after treatment with either R13 or CP11. No significant differences among the proportions of neurons expressing both TH and VGLUT1 that were retrogradely labeled from GAST or marked with both red and green tracers were found (Figure 5C: lower panels).

## 4. Discussion

Promising experimental treatments have aimed at enhancing the slow and inefficient process of axon regeneration after PNI via increased signaling through neuronal TrkB receptors, both using increased activity in injured neurons (Gordon and English, 2016) and pharmacological approaches (English et al., 2013, 2022; Isaacson et al., 2023). Both R13 and CP11 are candidates for such treatment for PNI. R13 metabolizes into a BDNF-mimetic, 7,8-DHF, and signals through the TrkB receptor. CP11 targets an enzyme, AEP, that is inhibited downstream of TrkB signaling (Zhang et al., 2014). In the current study, we aimed to investigate whether these treatments, that robustly enhance muscle sensory axon regeneration following PNI, also alter the composition of sensory axons successfully regenerating to reinnervate two different muscle targets with similar fiber type composition.

This study follows up on our previous observations that treatments with R13 or CP11 each enhanced the regeneration of axons of sensory neurons (English et al., 2022; Isaacson et al., 2023). Indeed, we found here that our treatments significantly increased the number of DRG neurons whose axons had successfully reinnervated two different muscles whose distinct innervation patterns are known to be disrupted following recovery from sciatic nerve transection and repair (English, 2005). The proportions of four classes of DRG neurons reinnervating these two muscles was evaluated in mice where sensory axon regeneration was clearly enhanced by our treatments. The classes studied were chosen because they represent different functional neuronal types: proprioceptive (VGLUT1), low threshold mechanoreceptive (TH), and peptidergic and non-peptidergic nociceptive (TRPV1 and IB4), and in combinations compatible with the use of available reagents used to mark them.

Our main finding was that proportions of the different neuronal classes in treated mice were found to be roughly the same as found in both vehicle-treated controls and intact animals. Of the 54 different possible comparisons made of the proportions found in reinnervated muscles and those of intact animals, only six resulted in significant differences. Among those significant differences, two were found in vehicle-treated mice and in both instances, treatments with either R13 or CP11 actually restored the proportions of phenotypes found in intact animals. Three significant changes were found in mice that had been treated with R13 (out of 18 comparisons) and only one in mice treated with CP11. Given the extent of misdirection of regenerating axons expected to reinnervate these muscles, we find this specificity of reinnervation remarkable. Of course, we have studied only four of the nine classes of DRG neurons, and only one subclass of the NF class. We also have restricted our analysis to afferent neurons reinnervating muscle targets. Future studies should aim to see if this remarkable specificity of reinnervation we have observed is also present in DRG neurons of other classes and reinnervating other targets. Following nerve transection and repair, significant misdirection of the regenerating axons is found. Peripheral targets are reinnervated by different axons than had done so prior to injury (Brushart, 1993, 2012; English, 2005; Gordon and English, 2016). This misdirection is exacerbated when regeneration is enhanced using low frequency electrical stimulation (English, 2005). We think it highly unlikely that our treatments stimulated the growth of regenerating neurites sufficiently to reduce or eliminate their misdirection. In fact, one might expect that the proportions of sensory neurons of different phenotypes that successfully reinnervated muscles after sciatic nerve transection and repair might be different from the native proportions, and that this would be especially true if regeneration was enhanced. How then might the findings of the present study be explained?

One explanation might be that regenerating axons selected their pathways. Successfully regenerating axons branch and enter one or more regeneration pathways in the distal segment of a cut and repaired nerve, but this takes place over a prolonged period after injury, referred to as “staggered axonal regeneration” (Brushart, 1993; Al-Majed et al., 2000). One interpretation of this delay is that it is associated with the initial selection of pathways in the distal segment of the repaired nerve by the regenerating axons. In addition, the branches of some early regenerating axons that had elongated into different regeneration pathways are later withdrawn, a second way that pathways might be selected (Brushart and Seiler, 1987; Witzel et al., 2005; Gordon and English, 2016). Indeed, (Bolivar et al., 2020) suggested that trophic factors, target contact, or potentially, repair Schwann cells that express certain markers that are retained even after regeneration, could influence how motor and sensory axons might choose to enter or remain in a pathway that leads them to specific targets. Similarly, Brushart proposed that specific Schwann cell markers associated with regeneration pathways might lead regenerating axons selectively to muscle or cutaneous targets (Brushart, 1988, 2012). This preferential reinnervation emerges only two or more weeks after nerve injury, a delay that might be consistent with pathway selection/retention by regenerating axons.

An alternative explanation for the specificity in reinnervation patterns observed here could stem from the response of the injured neurons to transcriptional reprogramming following a nerve injury. Axotomized sensory neurons stop expressing CTS genes and express a common suite of genes associated with an “injured state” and with axon regeneration. Once axon regeneration is successful, the CTS genes start to be re-expressed (Renthal et al., 2020). Irrespective of the class of a regenerating DRG neuron prior to injury, it might recover from this reprogramming to express a different set of CTS genes (and associated protein markers) than expressed prior to injury, through signaling from the target or a specific pathway that its regenerating axons are aligned with. Changes in phenotype in response to muscle- or pathway-specific signals could result in the successful regeneration of sensory neuronal classes in similar proportions regardless of treatment, as seen in our results. However, we cannot rule out that some of the specificity of muscle sensory reinnervation observed here may involve both of the proposed mechanisms. Further study into distinguishing the mechanism involved in this specificity will be necessary.

Despite our predominant finding that enhancing axon regeneration using either R13 or CP11 did not result in a significant change in the proportions of DRG neurons of the six phenotypes studied, relative to intact mice, some significant differences were found. A significant reduction in the proportion of PEP DRG neurons expressing the TrpV1 marker and an increase in that of NP neurons that bound IB4 was found in the reinnervated TA muscle in mice treated with R13. While such differences might be attributed to a differential effect of the R13 treatment on the post-injury survival of neurons of these classes, and therefore their availability for muscle reinnervation, our observation that similar changes were not found among DRG neurons reinnervating GAST would seem to rule out that notion. Similarly, we do not believe that the treatments with either R13 or CP11 that significantly increased the proportion of GAST sensory neurons immunoreactive for TH were the result of increased availability of TH+ neurons. Significant increases were not found among DRG neurons reinnervating TA. It is not clear currently why these differences occur in these classes of neurons and only in neurons reinnervating a particular muscle. It is possible that the differences observed are simply the result of unfinished responses to reprogramming or incomplete withdrawal of axons associated with pathway selection at the post-injury time (4 weeks) that we studied. Further investigation at longer times could be a goal of future studies.

## Data availability statement

The original contributions presented in the study are publicly available. This data can be found here: https://figshare.com/articles/dataset/Untitled_Item_Proportions_of_Four_Distinct_Classes_of_Sensory_Neurons_Are_Retained_Even_When_Axon_Regeneration_Is_Enhanced_Following_Peripheral_Nerve_Injury/24212361.

## Ethics statement

The animal study was approved by Institutional Animal Care and Use Committee of Emory University. The study was conducted in accordance with the local legislation and institutional requirements.

## Author contributions

SK: Conceptualization, Data curation, Formal analysis, Methodology, Visualization, Writing – original draft, Writing – review & editing. DC: Conceptualization, Data curation, Methodology, Supervision, Visualization, Writing – review & editing. RI: Data curation, Investigation, Methodology, Supervision, Writing – review & editing. AE: Conceptualization, Data curation, Formal analysis, Funding acquisition, Methodology, Project administration, Resources, Supervision, Validation, Visualization, Writing – original draft, Writing – review & editing.

## Glossary

**Table:**

7,8-DHF	7,8-dihydroxyflavone
AEP	asparaginyl endopeptidase (δ-secretase)
BDNF	brain-derived neurotrophic factor
BSA	bovine serum albumin
CP11	compound 11
CTS	cell type-specific
DRG	dorsal root ganglion
GAST	gastrocnemius muscle
IB4	Griffonia simplicifolia isolectin B4
i.p.	intraperitoneal
NF1	neurofilament high class of DRG neurons
NP	non-peptidergic unmyelinated primary afferent neurons
PBS	phosphate-buffered saline
PEP	peptidergic nociceptor neurons
PNI	peripheral nerve injury
R13	a 7,8-DHF prodrug
SD	standard deviation
TA	tibialis anterior muscle
TH	tyrosine hydroxylase
TrkB	tropomyosin receptor kinase B
TrpV1	transient receptor potential cation channel subfamily V member 1
VGLUT1	vesicular glutamate transporter 1
WGA	wheat germ agglutinin
